# Advancing Health Equity, Eliminating Health Disparities, and Improving Population Health

**DOI:** 10.5888/pcd18.210264

**Published:** 2021-08-12

**Authors:** Leonard Jack

**Affiliations:** 1 Office of Medicine and Science, National Center for Chronic Disease Prevention and Health Promotion, Centers for Disease Control and Prevention, Atlanta, Georgia

In June 2017, *Preventing Chronic Disease* (PCD) invited a panel of 7 nationally recognized experts in scientific publishing to respond to key questions about the journal’s mission, quality of scientific content, scope of operations, intended audience, and future direction (1). PCD and the panel of experts recognized that chronic disease is a major contributor to poor health outcomes, an increase in health care costs, and a reduction in quality of life. Reducing the burden of chronic disease is a challenge requiring diverse collaborations and dissemination and adoption of effective interventions in multiple settings. The expert panel strongly encouraged the journal to focus more on complementing its rich body of published work on epidemiological studies with content that is attentive to evaluating population-based interventions and policies.

Since its inception in 2004, PCD’s mission has been to promote dialogue among researchers, practitioners, and policy makers worldwide on the integration and application of research findings and practical experience to address health disparities, advance health equity, and improve population health. To better advance that mission, PCD used the panel’s recommendations to refine the journal’s focus, addressing 4 main areas of public health research, evaluation, and practice:

- Behavioral, psychological, genetic, environmental, biological, and social factors that influence health
- Development, implementation, and evaluation of population-based interventions to prevent chronic diseases and control their effect on quality of life, illness, and death
- Interventions that reduce the disproportionate incidence of chronic diseases among at-risk populations
- Development, implementation, and evaluation of public health law and health policy–driven interventions

Refining the focus on these 4 areas has allowed PCD to receive a wide range of content from authors around the world. In addition to manuscripts received through the journal’s regular submission process, PCD has issued calls for papers on topics that bring to the forefront timely public health issues and targeted public health responses to improve population health.

Advancing health equity and eliminating health disparities have been and continue to be critical factors to PCD in addressing these topic areas. Healthy People 2020 defines health equity as the attainment of the highest level of health for all people (2). According to Healthy People 2020, “Achieving health equity requires valuing everyone equally with focused and ongoing societal efforts to address avoidable inequalities, historical and contemporary injustices, and the elimination of health and health care disparities” (2). Healthy People 2020 defines health disparities as “a particular type of health difference that is closely linked with social, economic, and/or environmental disadvantage” (2).

As part of its mission to address these important issues, PCD is excited to release this collection, “Advancing Health Equity, Eliminating Health Disparities, and Improving Population Health.” Of the 17 articles in the collection, 10 were submitted in response to PCD’s call for papers for the collection and 7 were previously published in the journal. All articles underwent the journal’s rigorous peer-review process. In addition, this collection features a position statement on the journal’s commitment to advancing diversity, equity, and inclusion in its scientific leadership, peer review process, research focus, training, and continuing education (3).

Over the past decade, there has been a range of community-based, technically innovative, and clinically driven prevention strategies in public health to prevent and reduce the burden of chronic conditions among diverse populations worldwide. Articles in this collection describe innovative and successful work to address factors contributing to advancing health equity, eliminating health disparities, and improving population health. They provide the latest information on ways to better understand contextual factors responsible for influencing health outcomes (both negatively and positively) and effective approaches to improve population health among diverse populations in various settings. The 18 articles address these core themes from multiple perspectives:

1. PCD’s Commitment to Advancing Diversity, Equity, and Inclusion in Its Scientific Leadership, Peer Review Process, Research Focus, Training, and Continuing Education (3)
2. Engaging With Communities — Lessons (Re)Learned From COVID-19 (4)
3. Global Perspectives on Improving Chronic Disease Prevention and Management in Diverse Settings (5)
4. Reaching the Hispanic Community About COVID-19 Through Existing Chronic Disease Prevention Programs (6)
5. Community Engagement of African Americans in the Era of COVID-19: Considerations, Challenges, Implications, and Recommendations for Public Health (7)
6. Addressing Racial and Ethnic Disparities in COVID-19 Among School-Aged Children: Are We Doing Enough? (8)
7. A Framework for Mobilizing Health Care to Respond to the Community Within the COVID-19 Pandemic (9)
8. Addressing Emotional Wellness During the COVID-19 Pandemic: The Role of *Promotores* in Delivering Integrated Mental Health Care and Social Services (10)
9. COVID-19 and Chronic Disease: The Impact Now and in the Future (11)
10. Screening and Referral Care Delivery Services and Unmet Health-Related Social Needs: A Systematic Review (12)
11. Community and Research Perspectives on Cancer Disparities in Wisconsin (13)
12. Urban–Rural Disparities in Access to Low-Dose Computed Tomography Lung Cancer Screening in Missouri and Illinois (14)
13. Quantification of Potential Inequities in Breast Cancer Incidence in New Mexico Through Bayesian Disease Mapping (15)
14. HbA_1c_ Performance in African Descent Populations in the United States With Normal Glucose Tolerance, Prediabetes, or Diabetes: A Scoping Review (16)
15. Reducing Tobacco Use in Oregon Through Multisector Collaboration: Aligning Medicaid and Public Health Programs (17)
16. “We’re, Like, the Most Unhealthy People in the Country”: Using an Equity Lens to Reduce Barriers to Healthy Food Access in Rural Appalachia (18)
17. Oral Health Behaviors in Very Young Children in Low-Income Urban Areas in Chicago, Illinois, 2018–2019 (19)
18. A Randomized Trial to Improve Adherence to Follow-up Eye Examinations Among People With Glaucoma (20)

Positioning a scientific journal to address matters related to diversity, equity, and inclusion requires careful and intentional thinking and action. Going back to PCD’s inaugural issue in 2004, featuring an essay on social determinants of health by Dr Leonard Symes, professor emeritus of epidemiology at the University of California, Berkeley, the journal has created a space to highlight the importance of these topics in chronic disease prevention and health promotion. Since that first issue, PCD has continued to demonstrate a dedication to these issues at all levels: through its leadership and staff, the content it publishes, its expanding pool of talented volunteers (PCD’s external review panel, editorial board, associate editors, statistics review committee), the rigorous peer-review process, a comprehensive and inclusive variety of article types, calls for papers related to these issues, and more. In its 18 years of publication, PCD has consistently worked to assure the public of its commitment to achieving diversity, equity, and inclusion.

Peer-reviewed journals around the world are also focusing attention on these issues. In keeping with this movement, as PCD’s editor in chief I have authored the first article featured in this collection, which is a position statement on the steps already taken by the journal, steps planned for the next 5 years, and key measurable outcomes (3). PCD hopes to serve as a model in identifying and implementing best practices for diversity, equity, and inclusion to build an even stronger trust with the public.

And trust is needed: mistrust of the health care system has emerged as a primary barrier among members of communities of color to seeking care in health care systems (21). Mistrust stems from historical events, including the Tuskegee syphilis study, and is reinforced by health system issues and discriminatory events that continue to this day (21). This collection includes an article by Michener and colleagues, which posits that COVID-19 has underscored long-standing societal differences in drivers of health (4). The authors offer insights into this historical reality and suggest using a health equity lens to engage communities at risk of poor health outcomes, improve bidirectional communication, establish data sharing, and improve involvement in program implementation, dissemination, and evaluation. Authors share concrete ways these can be achieved by presenting successful examples around the US.

The global impact of COVID-19 among people at risk or living with a chronic condition in multicultural communities necessitates that health communication messages are created and delivered from a health equity perspective (22). Airhihenbuwa and coauthors, in their commentary, discuss the importance of culture in unpacking messages that may be the same globally (eg, physical/social distancing) yet different across cultures and communities (individualist vs collectivist) (5). Authors discuss how use of the PEN-3 framework can facilitate a community-engaged communication response to COVID-19.

Populations with low socioeconomic status and certain racial and ethnic groups (eg, Native American, Hispanic, and African American people) have historically been disproportionately affected by chronic disease, COVID-19 diagnosis, hospitalization, and mortality (23). Calo and associates discuss how COVID-19 has disproportionately affected Hispanic communities throughout the US (6). This commentary describes how Better Together REACH, a community–academic coalition promoting chronic disease prevention, and Project ECHO (a telementoring program based at Penn State University), were adopted to support a coordinated COVID-19 response in the Hispanic community in Pennsylvania. Authors provide insights into how the existing infrastructure of chronic disease programs can be used to leverage resources and provide trusted and continuous services to reach Hispanic populations during the pandemic.

African Americans, like the Hispanic population, are more likely to contract COVID-19, be hospitalized, and die of the disease (24). Akintobi et al describe how psychosocial, sociocultural, and environmental vulnerabilities, compounded by preexisting health conditions, exacerbate the burden of COVID-19 among African Americans (7). Authors share important information based on their years of experience on ways to create and implement approaches to intentionally engage African Americans at higher risk of COVID-19. Insights and recommendations can advance community leadership and be used to prepare public health practitioners, researchers, and evaluators for future pandemics — both assisting in advancing health equity and addressing historical aspects of health disparities among African Americans.

The disproportionate impact of COVID-19 and associated disparities among Hispanic, non-Hispanic Black, and non-Hispanic American Indian/Alaska Native children and teenagers has been widely reported (25). Children from some racial and ethnic minority groups have a higher prevalence of obesity, asthma, type 2 diabetes, and hypertension; were diagnosed more frequently with COVID-19; and had more severe outcomes compared with their non-Hispanic White counterparts (26). In addition, a higher proportion of children from some racial and ethnic minority groups, compared with White children, live in families with incomes less than 200% of the federal poverty level or in households lacking secure employment (8). White et al argue that the COVID-19 pandemic reemphasizes the importance of implementing policy, systems, and environmental changes in school systems to support emergency preparedness and recovery, as well as resilience, through collaborations among local health departments, local school systems, and other public and private organizations (8). Topics addressed in this article include disparities in underlying medical conditions and social determinants of health, inequities in social determinants of health, and community-based approaches to reducing COVID-19 disparities. The article concludes by discussing ways to implement strategies to advance health equity through partnership.

It has long been recognized that disparities in health care access and patient outcomes are associated with factors related to race, sex, gender, sexual orientation, primary language, and socioeconomic status (27). Epps and coauthors recognize that African Americans and other underrepresented racial and ethnic groups are often not included in health decision making and policy development (9). As a result, these public health experts describe steps undertaken to improve participation, joint decision making, and capacity building between an integrated academic health system and a community coalition to address complex health challenges with the aim of increasing the capacity of health systems to reduce the burden of COVID-19. This article describes a call to action by the chair of a health care board of trustees to its board members consisting of clinicians, researchers, educators, and health advocates to identify ways to mitigate disparities and determine how the health care system could play a role in advancing and implementing effective strategies to reduce the disproportionate burden of COVID-19 among communities of color. Authors provide insight into the organizational planning process to generate a community outreach and health disparities collaborative with goals for governance, messaging and education, community partnerships, data, and research and evaluation.

COVID-19 has exacted a tremendous toll on the physical, emotional, and psychological well-being of many Americans, thus requiring a population health response (28). The disproportionate impact of the COVID-19 pandemic on Hispanic communities has resulted in a greater burden of depression, anxiety, and stress along with the need for increased assistance with housing, access to food, and supplemental income (29). Moon and colleagues offer original research that reports findings on demographic characteristics and factors associated with service volume, types of services, and referrals in the pre-COVID-19 and COVID-19 periods (10). They report that referrals shifted from primarily mental health services and disease management during the prepandemic period to affordable housing support, food assistance, and supplemental income during the COVID-19 period. This study presents findings on how a community-based organization with a long-standing presence in the Hispanic community effectively expanded its emotional wellness program, using *promotores* to provide integrated mental health care and social services to clients disproportionately affected by COVID-19.

Hacker et al discuss the problem of COVID-19 and chronic disease in their essay (11). They describe 3 categories of challenges facing public health professionals and identify solutions needed to improve health outcomes and lessen health inequities among people at risk or living with a chronic disease. Authors also discuss the evolving response by the Centers for Disease Control and Prevention’s National Center for Chronic Disease Prevention and Health Promotion to implement a multipronged approach to enhance access to data at the local level, focus on addressing social determinants of health through a health equity lens, and expand partnerships and communication about the impact of COVID-19 on chronic disease.

Unmet health-related social support needs among people being served by health care systems can contribute to high patient morbidity and poor population health (30,31). However, little is known about the overall impact of screening and referral programs that address unmet health-related social needs on outcomes related to experience of care, population health, and cost. Ruiz Escobar et al conducted a systematic review of peer-reviewed articles in PubMed published over the past 10 years (as of March 2020) to determine the impact of screening and referral care delivery services on unmet health-related social needs (12). Thirty-five articles met the systematic review’s inclusion criteria. After conducting their review, the authors concluded that although evidence exists of a positive influence of screening and referral program outcomes related to experience of care and population health, no definitive conclusions could be made on the overall impact on changes in patient connection to resources, patient satisfaction, and patient-reported outcomes because of the potential high risk of bias across studies. Their findings can inform the use of screening and referral programs in health care organizations, including ways to strengthen future studies to examine their effectiveness.

Qualitative research is an important methodological tool that provides critical insights in identifying subjective meaning in the context of health (32). Qualitative research is a necessary exploratory approach that can be used to better understand and improve health equity research and practice. Olson and her team of researchers conducted 10 listening sessions and 28 interviews with people from diverse backgrounds to identify themes in causes, solutions, and opportunities to collaborate across sectors to address cancer disparities (13). Researchers validated the use of qualitative approaches to engage diverse participants representing many different sectors. Qualitative findings identified medical mistrust, the need for equitable multilevel partnerships, influences of environmental threats on cancer burden, and location of cancer disparities as key concerns among people participating in the listening sessions and interviews. The researchers describe how these findings will be used to form multisector teams to address local social, cultural, and biological influences of cancer disparities and achieve health equity in Wisconsin.

Geographic location continues to be an important contributor in shaping access to timely and necessary screening and treatment options (33). Rohatgi et al conducted original research examining relationships among rurality, sociodemographic characteristics, and access to low-dose computed tomography (LDCT) screening for lung cancer and screening access and lung cancer mortality (14). This study revealed that more than 97% of metropolitan residents had access to LCDT screening, compared with just over 40% of nonmetropolitan residents. Researchers learned that residents of southeastern Missouri, a rural and impoverished area, had low screening access, high smoking prevalence, and high lung cancer mortality. Researchers concluded that targeted strategies to implement rural LDCT screening could reduce geographic disparities in access, and future research could help identify factors that increase access to screening to eliminate rural-related disparities in lung cancer mortality.

Breast cancer is the most frequently diagnosed cancer and a leading cause of cancer mortality among American Indian/Alaska Native (AI/AN) women (34). Despite having a lower incidence of breast cancer than White women, AI/AN women are more likely to be diagnosed at younger ages and later stages (35). Breast cancer incidence among non-AI/AN women has largely been quantified in large geographic regions in the US, and substantial regional variation in breast cancer inequities in non-Hispanic AI/AN populations has been reported. Zahrieh and colleagues conducted research to obtain a deeper understanding at a granular level to identify potential inequities in breast cancer incidence by applying county-level Bayesian disease mapping (a model-based approach that offers a means to improve county-level incidence estimates) to population surveillance data from 2005 through 2014 in New Mexico (30). They found a significant overall disparity effect across New Mexico, evidenced by the age-adjusted rate of breast cancer among non-Hispanic AI/AN women being appropriately 0.38 times the corresponding age-adjusted rate among non-Hispanic White women. Researchers also suggest that findings can be used to facilitate targeted statewide and county-level cancer control interventions to mitigate breast cancer disparities among AI/AN women in New Mexico.

Historically, type 2 diabetes has disproportionately affected racial and ethnic minority groups (31). To ensure accurate detection of type 2 diabetes, we must understand the ability of hemoglobin A_1c_ (HbA_1c_) to correctly classify type 2 diabetes status and evaluate intra-ethnic variation. Toward this end, Khosla et al conducted a scoping review to determine HbA_1c_ performance in African descent populations in the US with normal glucose tolerance, prediabetes, and diabetes (16). Results included 7 studies that analyzed HbA_1c _performance among African Americans, 1 study that analyzed HbA_1c_ performance in Afro-Caribbean people, and 4 studies that analyzed HbA_1c _performance among Africans. Researchers found that current HbA_1c_ cutoffs for prediabetes and type 2 diabetes may overestimate glycemic status in African Americans and underestimate glycemic status in Afro-Caribbean and African people. Researchers indicated that alternating testing, such as the oral glucose tolerance test, fasting plasma glucose, and other glycated blood proteins in place of or in combination with HbA_1c _may better assess glycemic status in populations of African descent.

Tobacco use remains the leading cause of preventable morbidity and mortality in the US (33). Livingston and colleagues evaluated changes in tobacco cessation benefits, patient access, and cigarette smoking prevalence before and after 16 coordinated care organizations began providing comprehensive cessation benefits for reducing tobacco use prevalence among Medicaid members in Oregon (17). This implementation evaluation identified changes in tobacco cessation benefits, patient–provider discussions of smoking cessation, and cigarette smoking prevalence before and after the introduction of statewide incentives for reducing cigarette smoking. Evaluators reported that statewide effort accelerated progress toward tobacco use reduction among members of coordinated care organizations.

Obesity among adults living in Appalachia continues to be a major problem, and policy, systems, and environmental interventions may help to address long-standing underlying factors that have historically contributed to this persistent public health concern (35). Cardarelli and associates reported findings from a qualitative study that used a grounded theory approach to identify barriers and facilitators for healthy food access in a rural county in Kentucky (18). The goal was to design interventions responsive to social, cultural, and historical contexts from an equity perspective. Focus group participants were asked, for example, if it was easy to get fruits and vegetable at locations where they purchase food, if many people in their community purchase food at farmers markets, and what factors in their community make it easier or harder to eat healthy. The authors concluded that efforts to address food access through policy, systems, and environmental interventions must be sensitive to characteristics of the rural setting, acknowledge social inequities in the region, and proactively engage community members throughout all stages of intervention planning, implementation, and evaluation.

Oral health disparities among children have been linked to socioeconomic inequalities, access to care, health systems barriers, and lack of access to foods that promote optimal oral health outcomes (39). Martin et al conducted original research that explored the frequency of tooth brushing among children with a mean age 21.5 months (19). Their results indicated that the frequency of brushing among children, as reported by guardians, was higher when the correct amount of toothpaste was used, brushing occurred for a longer duration, and other family members helped children with brushing. Their findings strongly suggest that parental and family support for brushing are critically important in promoting and sustaining tooth-brushing behaviors.

According to the *Lancet *Global Health Commission on Global Eye Health, women, rural populations, and racial/ethnic minority groups are more likely to have vision impairment, a pervasive inequality that needs to be addressed (40). This PCD collection on advancing health equity and reducing health disparities concludes with a research study by Leiby et al investigating the effectiveness of an enhanced intervention among people with glaucoma: using patient navigators and social workers to improve patient adherence to follow-up eye care in community settings (20). The study compared the intervention group with a group of patients in usual care. Participants in usual care were provided with a local ophthalmologist’s contact information and a copy of their eye examination results; they were not provided access to patient navigators or social workers. Study participants, who were randomly assigned to either the enhanced or usual care intervention, were a diverse group of participants aged over 40 with a family history of glaucoma or currently diagnosed with diabetes. Only participants who had not seen an ophthalmologist in the previous 12 months were permitted to enroll in the study. Study participants consisted largely of African Americans, followed by White, Asian American, and Hispanic residents of Philadelphia, Pennsylvania. The study found that the use of patient navigators and social workers doubled the rate of adherence to annual recommended eye care follow-up, compared with participants assigned to the study’s usual care intervention. The study highlights that formalized use of social support in partnership with local ophthalmologists can be an effective approach to increasing access to local ophthalmological services.

At the center of this collection of articles is a shared commitment to the goal of eliminating health disparities, particularly those that continue to persist despite aggressive efforts to ameliorate them. The collection describes a range of diverse and timely examples of efforts to eliminate health disparities and advance health equity among racial and ethnic groups in the US. Articles appearing here represent various types of PCD articles that encompass multiple perspectives, from original research and systematic reviews to implementation evaluation to expert commentaries to tools that can be used in public health practice. As a discipline, we have important work to do, not only to better understand how social determinants of health and other contextual factors impact health but also to design, implement, and evaluate effective multilevel systems approaches that create optimal conditions to promote health for all. PCD will continue to move forward in its commitment to these goals, and we encourage authors to visit the journal’s Author’s Corner website (https://www.cdc.gov/pcd/for_authors/index.htm) to learn more about article types that best fit their research addressing population-based approaches to eliminating health disparities and advancing health equity.
